# The protein interaction network of a taxis signal transduction system in a Halophilic Archaeon

**DOI:** 10.1186/1471-2180-12-272

**Published:** 2012-11-21

**Authors:** Matthias Schlesner, Arthur Miller, Hüseyin Besir, Michalis Aivaliotis, Judith Streif, Beatrix Scheffer, Frank Siedler, Dieter Oesterhelt

**Affiliations:** 1 Department of Membrane Biochemistry, Max Planck Institute of Biochemistry, Martinsried, Germany; 2 Division of Theoretical Bioinformatics (B080), German Cancer Research Center (DKFZ), Heidelberg, Germany; 3 Protein Expression & Purification Core Facility, EMBL Heidelberg, Germany; 4 Institute of Molecular Biology and Biotechnology, FORTH, Heraklion, Crete, Greece; 5 Institute of Microbiology, Technische Universität Braunschweig, Braunschweig, Germany; 6 Department of Cellular and Molecular Biophysics, Max Planck Institute of Biochemistry, Martinsried, Germany

## Abstract

**Background:**

The taxis signaling system of the extreme halophilic archaeon *Halobacterium (Hbt.) salinarum* differs in several aspects from its model bacterial counterparts *Escherichia coli* and *Bacillus subtilis*. We studied the protein interactions in the *Hbt. salinarum* taxis signaling system to gain an understanding of its structure, to gain knowledge about its known components and to search for new members.

**Results:**

The interaction analysis revealed that the core signaling proteins are involved in different protein complexes and our data provide evidence for dynamic interchanges between them. Fifteen of the eighteen taxis receptors (halobacterial transducers, Htrs) can be assigned to four different groups depending on their interactions with the core signaling proteins. Only one of these groups, which contains six of the eight Htrs with known signals, shows the composition expected for signaling complexes (receptor, kinase CheA, adaptor CheW, response regulator CheY). From the two *Hbt. salinarum* CheW proteins, only CheW1 is engaged in signaling complexes with Htrs and CheA, whereas CheW2 interacts with Htrs but not with CheA. CheY connects the core signaling structure to a subnetwork consisting of the two CheF proteins (which build a link to the flagellar apparatus), CheD (the hub of the subnetwork), two CheC complexes and the receptor methylesterase CheB.

**Conclusions:**

Based on our findings, we propose two hypotheses. First, *Hbt. salinarum* might have the capability to dynamically adjust the impact of certain Htrs or Htr clusters depending on its current needs or environmental conditions. Secondly, we propose a hypothetical feedback loop from the response regulator to Htr methylation made from the CheC proteins, CheD and CheB, which might contribute to adaptation analogous to the CheC/CheD system of *B. subtilis*.

## Background

Taxis, the directed movement along gradients towards more favorable locations, is widespread among Bacteria and Archaea. Whereas the motility apparatus is different in Archaea and Bacteria [1,2], the two-component signal transduction system controlling it to direct tactic movements is—with some variations—conserved throughout all prokaryotes [3].

The archaeon *Halobacterium (Hbt.)salinarum* offers a great opportunity for studying taxis signal transduction without time lag after fine-dosed addition and removal of stimuli because of its phototactic capability [4]. The taxis signal transduction system of *Hbt.salinarum* is with respect to its protein inventory more similar to the more complex system of *B.subtilis* than to the streamlined system of *E.coli*[3,5,6]. Functionally, however, this is not true in every respect. For example, CheA in *Hbt.salinarum* is activated by repellent stimuli [7], which is similar to that of *E.coli*[8] and different from that of *B.subtilis*[9].

*Hbt.salinarum* genome codes for ten homologues of bacterial Che proteins and two archaeal CheF proteins [5,6,10]. *CheF1*, *cheF2*, *cheR*, *cheD*, *cheC1*, *cheC3*, *cheB*, *cheA*, *cheY*, and *cheW1* are organized into one gene cluster (http://www.halolex.mpg.de/; [11]). A second *cheW* homologue, *cheW2*, is located close to the *fla* gene region (the **fl**agella **a**cessory genes are required for flagella assembly and function [12-15]). A third *cheC*, *cheC2*, is located elsewhere in the genome. Table 1 gives an overview about the *Hbt.salinarum* Che proteins and their function.

**Table 1:** **Functions of the Che proteins of*Hbt.salinarum***

**Protein**	**Demonstrated functions in *Hbt.salinarum***	**Demonstrated functions of holomogues in other organisms**
CheA	Phosphorylation of CheY [16]	Phosphorylation of CheY and CheB [17,18]
CheW1		Coupling of CheA to receptors [19]
CheW2		Coupling of CheA to receptors [19]
CheY	Essential for switching and	Switching/CCW (CW) rotation in Bsu (Eco) [20-22]
	CCW swimming [7]	
CheB	Receptor demethylation and	Receptor demethylation [23,24]; in Eco also
	deamidation [25]	deamidation [26]
CheR	Receptor methylation [25]	Receptor methylation [23,27]
CheC1		CheY-P phosphatase [28], CheD inhibition [29,30]
CheC2		CheY-P phosphatase [28], CheD inhibition [29,30]
CheC3		CheY-P phosphatase [28], CheD inhibition [29,30]
CheD		Receptor deamidase and enhancer of CheC in Bsu
		[30,31], receptor deamidase and methylesterase in
		Tma [32]
CheF1	Coupling Che system to	
	archaeal flagellum [10]	
CheF2		

Functions in other organisms are thought to be universal, unless certain organisms are indicated (Eco: *E.coli*, Bsu: *B.subtilis*, Tma: *T.maritima*).

Furthermore, 18 homologues to eubacterial methyl-accepting chemotaxis proteins (MCPs) have been identified [5,6]. These so-called halobacterial transducers (Htrs) either include their own sensing domain so that they act as receptors and transducers in one molecule or they interact with separate receptor proteins [33,34]. Six of the Htrs were predicted to contain no transmembrane domain and are assumed to recognize intracellular signals. The other Htrs contain two or more transmembrane helices and recognize signals at the membrane or extracellularly. The function of only eight Htrs has been assigned to-date (Table 2).

**Table 2:** The halobacterial transducers as preys

**Htr**	**Gene**	**Name**	**Signal**	**TM**	**A**	**Y**	**W1**	**W2**	**R**
1	OE3347F	HtrI	Orange light (A), UV light (R) [35-37]	2	∙	∙	∙	∙	
2	OE3481R	HtrII	Blue light (R), Ser (A) [38,39]	2	∙	∙	∙	∙	
3	OE3611R	BasT	Leu, Ile, Val, Met, Cys (A) [33]	2	∙	∙	∙	∙	
4	OE2189R	Htr4		2	∙	∙	∙	∙	
5	OE3474R	CosT	Compatible osmolytes (A) [34]	2	∙	∙	∙	∙	
6	OE2168R	Htr6		2	∙	∙	∙	∙	
8	OE3167F	HtrVIII	*O*_2_ (A) [40]	6	∙	∙	∙	∙	
14	OE1536R	MpcT	ΔΨ (A) [41]	2	∙	∙	∙		
17	OE3436R	Htr17		3	∙	∙			
18	OE2195F	Htr18		2	(∙)	∙			
16	OE1929R	Htr16		2	∙				
15	OE2392R	Htr15		0		∙	∙	∙	
11	OE5243F	Car	Arg (A) [42]	0				∙	
13	OE2474R	Htr13		0			∙	∙	
12	OE3070R	Htr12		0					∙
7	OE3473F	Htr7		3					
9	OE2996R	Htr9		0					
10	OE3150R	HemAT	*O*_2_ (R) [43]	0					

Transducers were grouped according to their interaction patterns. Signal indicates attractant (A) or repellent (R) signal for the respective transducer where known. TM is the number of predicted transmembrane helices. The columns A, Y, W1, W2 and R indicate whether the transducer was identified as interaction partner CheA, CheY, CheW1, CheW2 or CheR, respectively. () Htr18 was not identified with the bait CheA but its putatively associated protein OE2196F.

While the confirmed processes in *Hbt.salinarum* taxis signaling have already led to modeling of motor switching and signal processing [44-47], the understanding on a molecular level is still far from complete. For example, it is still unknown why *Hbt.salinarum* possesses more than one homologue of CheW, CheC and CheF. The function of CheD and the CheC proteins, which build one of the three adaptation systems in *B.subtilis*[48], is unclear in *Hbt.salinarum*. The mechanism of action of the switch factor fumarate, which was discovered in *Hbt.salinarum* 20 years ago [49,50], is also unresolved.

Because classical approaches to define function, for example deletion mutant analysis, are not always conclusive, we set out to investigate the taxis signal transduction system of *Hbt.salinarum* by protein interaction analysis. In the course of this study, we identified and characterized the archaeal chemotaxis protein family CheF that connects the bacterial-like taxis signaling system to the archaeal flagellar apparatus [10]. Here we report the interaction network of the *Hbt.salinarum* taxis signaling proteins which presents new knowledge about established Che proteins and identifies connections to proteins that were not known to be linked to taxis signal transduction.

## Results and Discussion

### Protein-protein interaction analysis in *Hbt.salinarum*

Like all halophilic archaea, *Hbt.salinarum* balances the osmotic pressure of its environment by accumulating up to 5 M salt (mainly KCl) in the cytoplasm [51]. Haloarchaeal proteins are adapted to these conditions: they contain an excess of acidic amino acids, especially on the surface of the protein, and the frequency of the basic amino acid lysine is reduced [52,53]. While maintaining solubility and stability under high-salt conditions, the adapted proteins tend to lose their physiological interactions and even denature in solutions of low ionic strength (see [54] and references therein).

At the beginning of this study we were not aware of any method that had been successfully applied to analyze the interactions between halophilic proteins on a medium or large scale. Screening a test set of expected interactors from *Hbt.salinarum* using the yeast two-hybrid system failed for all tested haloarchaeal proteins (data not shown). The reason turned out to be autoactivation by the (acidic) *Hbt.salinarum* proteins being used as bait and probably also misfolding of the halophilic proteins when expressed in yeast.

To circumvent these issues, we established two affinity purification methods for haloarchaeal protein complexes with subsequent identification of the complex components by mass spectrometry (affinity purification mass spectrometry, AP-MS). As demonstrated earlier, the cellulose-binding domain (CBD) from the CipB protein from *Clostridium thermocellum* can be used as an affinity tag to purify halophilic proteins under high salt conditions [55-57]. We expressed the proteins under investigation—which were then called bait proteins—fused to this salt-insensitive affinity tag in their native host *Hbt.salinarum* to ensure correct folding of the halophilic proteins (Additional file 1). We put the bait proteins under control of a relatively strong promoter resulting in bait overproduction. This was necessary to overcome sensitivity problems but came at the cost of losing the cellular stoichiometry between the bait protein and its interaction partners.

**Figure 1:**
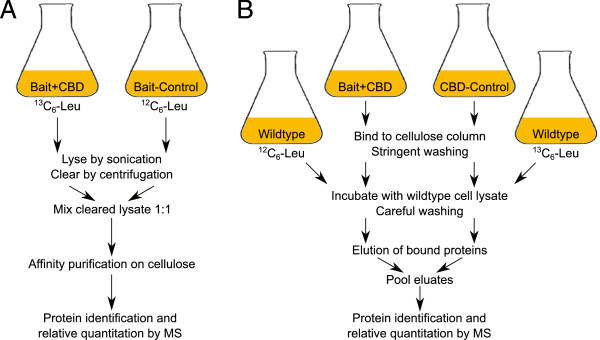
**Schematic of purification procedures. A** One-Step bait fishing. A *Hbt.salinarum* strain overexpressing the bait protein fused to CBD is cultured in synthetic medium containing ^13^C_6_-leucine. The corresponding bait-control strain overexpressing the bait protein without CBD is cultured in synthetic medium containing ^12^C_6_-leucine. The lysate from both strains is mixed and purification done on one cellulose column. **B** Two-Step bait fishing. The bait overexpression strain and a general CBD-control strain expressing plain CBD are grown in complex medium. The bait-CBD fusion and the plain CBD are bound to separate cellulose columns and stringently washed to remove all proteins except bait or CBD. The columns are incubated with lysate from *Hbt.salinarum* cells grown in synthetic medium containing ^12^C-leucine (bait) or ^13^C-leucine (pMS4), respectively. After elution, the eluates are pooled.

In our first method, termed one-step bait fishing (Figure 1A), *Hbt.salinarum* cells expressing the bait-CBD fusion protein were lysed and the cell lysate was applied to a cellulose column. This enabled binding of the bait protein along with its endogenous protein interaction partners (the prey proteins) to the column. After careful washing to remove unbound proteins, the bait-prey complexes were eluted from the column and proteins identified by mass spectrometry.

To discriminate specific interaction partners from nonspecific binders, we combined the purification procedure with stable isotope labeling by amino acids in cell culture (SILAC) [58,59]. For this, a second *Hbt.salinarum* strain which expresses the bait protein under the same strong promoter as in the bait-CBD strain but without CBD fusion, the bait-control strain, was used. Both strains were treated equally with the exception that the bait-CBD strain was grown in medium containing ^13^C_6_-leucine while the bait-control strain was grown in medium containing ^12^C_6_-leucine. Lysates from both strains were pooled and affinity purification was done from the pooled lysate. Finally, the ratio between the relative amount of the ^12^C-form and the ^13^C-form of the identified proteins (the SILAC ratio) was determined. To allow easier visualization, a symmetrical measure, called association score, was calculated from the SILAC ratio as described in the methods section. The association score indicates if an identified protein was specifically enriched by binding to the respective bait: in case of a specific interactor mainly the ^13^C-form would be present in the eluate, whereas for unspecific binders the ^13^C- and the ^12^C-form would be present to nearly the same extent. Proteins with an association score greater than seven were considered to be interactors and all other proteins to be nonspecific binders (for details see Additional file 2).

In our second method, two-step bait fishing (Figure 1B), lysates from the bait-CBD strain and a CBD-control strain (which expresses the plain CBD under the same promoter used for the bait-CBD fusions) were applied to separate cellulose columns. A stringent washing step followed which removed (nearly) all bound proteins except the bait-CBD fusion protein or the CBD, respectively. The bait-CBD loaded cellulose column was then incubated with lysate from *Hbt.salinarum* wildtype cells grown with ^12^C_6_-leucine, while the CBD-loaded column was incubated with lysate from *Hbt.salinarum* wildtype cells grown with ^13^C_6_-leucine. After careful washing to remove unbound proteins, the bait-prey complexes which formed on column were eluted, the eluates pooled, and proteins identified by mass spectrometry. Determination of the association score to discriminate specific and unspecific binders was done as for one-step bait fishing. In two-step bait fishing, the SILAC labeling was reversed compared to one-step bait fishing. This was necessary to account for strong interactors of the bait protein which could not be completely removed in the stringent washing step before incubation with wild type cell lysate (Figure 1B). This residual prey protein, which is ^12^C-labeled because the bait for two-step fishing is expressed in complex medium, would otherwise lead to erroneously low or even negative association scores.

**Figure 2:**
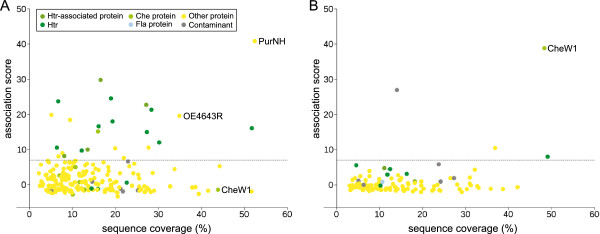
**Comparing one-step and two-step bait fishing using the bait CheA as an example.** The association score of the identified proteins is plotted against the sequence coverage with which the prey protein was identified. The dashed line indicates the threshold used in this study for assuming an interaction. For the underlying data see Additional file 3 and Additional file 4. **A** One-Step bait fishing. Several Htrs along with their associated proteins as well as the novel interactors PurNH and OE4643R were identified with high association scores. However, the association score for the expected interactor CheW1 is almost 0, which means the SILAC ratio was close to 1, even though this prey was identified with an unusually high sequence coverage. This indicates an enrichment by CheA. **B** Two-Step bait fishing. Here the interaction with CheW1 is clearly identified, whereas the interactions with the Htrs and with PurNH and OE4643R, which were later confirmed with these proteins as bait, are not detected. PurNH, OE4643R and several Htrs were not even identified, which indicates no or at least much weaker enrichment of these proteins in two-step bait fishing compared to one-step bait fishing.

When assessing the methods, we found that in most cases one-step bait fishing allowed a clear differentiation between specifically enriched proteins (which were then considered to be interaction partners) and the vast majority of background proteins through the association score. However, in a few cases, certain expected interaction partners showed an association score close to zero in one-step bait fishing (e. g., CheW1 copurified with CheA, Figure 2A). This was even more surprising since these proteins were identified with very high sequence coverage (the percentage of the protein sequence covered by matching peptides) with the corresponding baits (and with very low sequence coverage or not at all with other baits), which indicates specific enrichment. The reason for this is probably exchange of the prey protein from the bait-CBD lysate and the bait-control lysate in the short time (2–3 minutes) between mixing the lysates and washing unbound proteins away.

With two-step bait fishing, the CheA-CheW1 interaction could be clearly demonstrated (Figure 2B). In contrast, the interactions of CheA with Htrs as well as the novel interactors PurNH and OE4643R (discussed below), which were identified by one-step bait fishing, were missed in the two-step experiment. Hence both methods miss certain interactions which can be detected by the other method.

Aside from affinity, the properties determining the detectability of an interaction by one-step or two-step bait fishing are mainly the association and dissociation kinetics. The kinetics vary over several orders of magnitude for biologically relevant protein-protein interactions [60], and a broad spectrum of kinetics has been observed for the interactions of chemotaxis signaling proteins [61]. It can be expected that one-step bait fishing is effective for interactions with slow kinetics—here termed static interactions—whereas it will miss interactions with fast kinetics, which we call dynamic interactions. However, if the affinity is sufficiently high, dynamic interactions should be detectable by two-step bait fishing. On the other hand, two-step bait fishing will probably miss static interactions, because the exogenously added bait might not be able to displace its already bound endogenous counterpart. Detection of interactions by both one-step and two-step bait fishing can occur if either the interaction is of low dynamics resulting in enough stability for detection by one-step bait fishing but allowing enough exchange for prey binding to the exogenously added bait in two-step bait fishing, or if the interaction is static but prey protein with free bait binding sites is present in wild type cells and thus accessible to the exogenously added bait in two-step bait fishing.

As a further difference, in two-step bait fishing the prey proteins are purified from genetically unmodified cells, which excludes effects of chromosomal integration of the tagging vector at the locus of the bait protein upon the expression of interaction partners. This might be of particular importance as interacting proteins are often located adjacent to each other in the genome or even in one operon [62].

Since the methods detect different subsets of interactions, we applied both of them to all proteins under investigation. A similar strategy, the combination of MAP (mixing after purification)-SILAC and PAM (purification after mixing)-SILAC was developed by Wang and Huang [63] and demonstrated to outperform standard SILAC experiments for the identification of protein interactions with a broad range of kinetics.

### Interaction analysis of the *Hbt. salinarum* taxis signal transduction system

Initially, the interactions of the ten known *Hbt.salinarum* Che proteins were analyzed. Afterwards six additional proteins that were found to be interaction partners were used as baits to confirm the detected interactions and to extend the interaction network (Additional file 5).

Overall, the experiments resulted in 5505 reliable protein identifications (ProteinProphet [64]; probability > 0.95), detecting 597 unique proteins (Additional file 3). Of the identifications made, 267 were classified as interactions. Applying the spoke model [65] to derive binary interactions from the copurification data resulted in a final set of 201 unique interactions.

**Figure 3:**
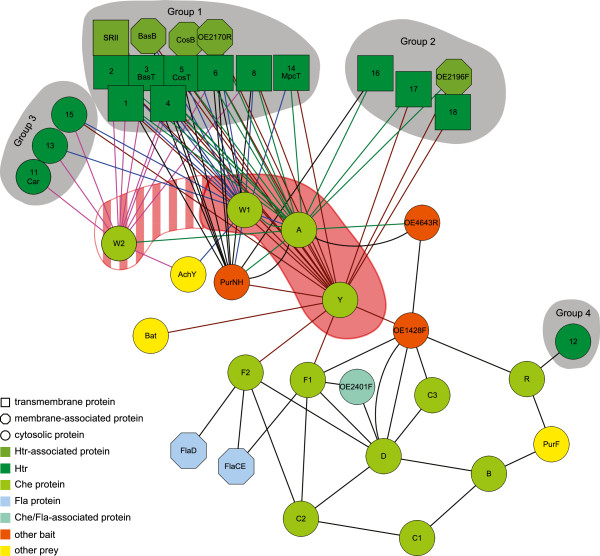
**Chemotaxis protein interaction network.** The spoke model was used to derive binary interactions from the copurification data. Only proteins discussed in the text are shown. The complete network is depicted in Additional file 6. The prefixes “Che” and “Htr” were omitted from the protein labels. The core signaling proteins CheA, CheW1 and CheY are highlighted by red shading. The weak binding of CheW2 to the core signaling complexes (see text) is indicated by red and white stripes. The gray areas delineate different groups of Htrs that can be distinguished by their interactions with CheA, CheR, CheW1, CheW2 and CheY (see text). For clarity, interactions identified with these baits are shown in different colors.

The resulting interaction network is depicted in Figure 3. For the sake of clarity, only interactions discussed in the text are included. The complete network is available from Additional file 6.

**Figure 4:**
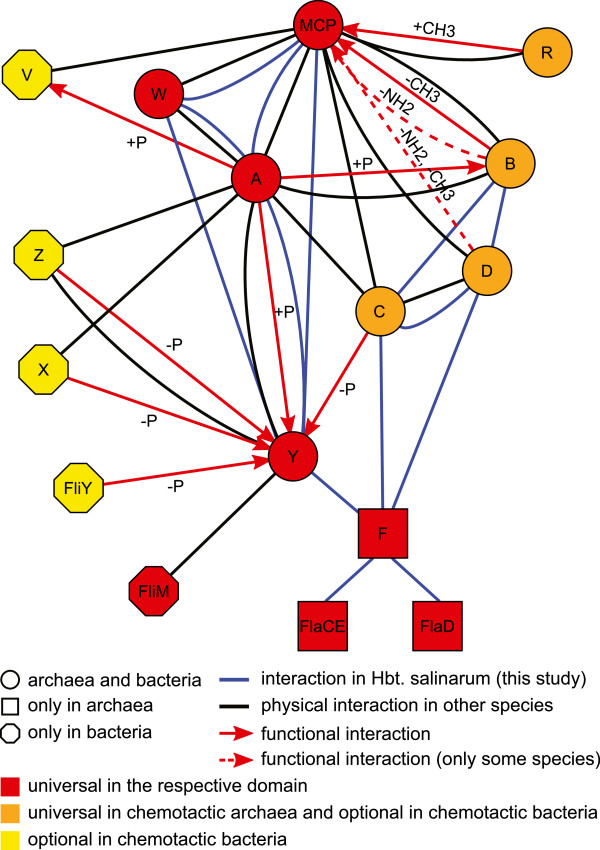
**Physical and functional interactions in prokaryotic taxis signaling systems.** The interactions of the core signaling proteins are generally in agreement between *Hbt.salinarum* and the data of the other organisms. The *Hbt.salinarum* dataset probably contains indirect interactions (e. g. CheY-CheW, CheY-Htr) because it was generated by AP-MS. The interactions of the other Che proteins have, with the exception of CheC-CheD, not been described in other organisms. References for literature data are given in Additional file 7.

The interactions detected in this study were compared to interactions between the Che proteins in other prokaryotic organisms (Additional file 7). However, the comparability of the datasets is rather low because the only other protein-protein interaction (PPI) study in an archaeal organism (*P.horikoshii*, [66]) reported just one interaction between Che proteins (CheC-CheD). The large-scale studies in bacteria (*Escherichia coli*[67,68], *Helicobacter pylori*[69], *Campylobacter jejuni*[70], *Treponema pallidum*[71]) as well as a dedicated PPI study of the *E.coli* taxis signaling system [72] were performed in organisms with quite different taxis signaling systems compared to that of *Hbt.salinarum*. For example, none of these organisms contains CheC and CheD proteins, which together account for a substantial part of the interactions described in the present study. Figure 4 presents a general interaction network for prokaryotic taxis signaling systems.

### The core signaling structure

The centerpiece of the chemotaxis signal transduction system is the histidine kinase CheA, which is bound to the Htrs together with the coupling protein CheW. It phosphorylates the response regulator CheY to generate the output signal CheY-P [19,73].

Bait fishing experiments with the core signaling proteins confirmed this assumed organization of the core structure (Figure 3) and also led to the identification of novel protein complexes around the core signaling proteins (described below). CheA was found to strongly interact with CheW1, and 6 of the 18 Htrs were found to interact with both CheA and CheW1. The putative associated proteins sensory rhodopsin II (SRII), BasB, CosB, OE2170R, and OE2196F (the latter two are putative periplasmic substrate binding proteins like BasB and CosB) were copurified with the Htrs 2, 3 (BasT), 5 (CosT), 6 and 18. Both CheA and CheW1 as well as several Htrs were detected as interaction partners of CheY. It should be emphasized that AP-MS analysis does not reveal the exact complex topology, so the interactions between CheY and CheW1 or the Htrs might be indirect via CheA. Details about the interactions of the core signaling proteins are presented in the following section.

### Different groups of Htrs can be distinguished by their interactions

In several prokaryotic organisms taxis receptors assemble into large, mixed clusters [74-81] which facilitate signal integration, large signal amplification and high sensitivity [76,82-85]. Due to this cluster formation it is not possible to deduce whether certain Htrs directly interact with a Che protein from copurification experiments.

Nevertheless, several conclusions about the interactions of the Htrs can be drawn from our data. The 18 Htrs of *Hbt.salinarum* show different patterns of interactions when all experiments are compared (Figure 3 and Table 2). According to their interactions, the Htrs can be classified into four groups: (1) the membrane-bound Htrs 1, 2, 3, 4, 5, 6, 8 and 14 were fished by CheW1, CheA and CheY and, with the exception of Htr14, also by CheW2. Six of the eight Htrs with known signals fall into this group; (2) the membrane-bound Htrs 16, 17 and 18 were copurified with CheA and CheY but with none of the CheWs; (3) the cytosolic Htrs 11, 13 and 15 were fished by CheW2 and to lesser extent also by CheW1 (except Htr11). They were not fished by CheA and, with the exception of Htr15, by CheY; and (4) Htr12 was fished only with CheR. Htrs 7, 9 and 10 did not interact with any Che protein (but they were identified by our MS method in some experiments and were therefore present in the cell and potentially identifiable) and thus cannot be assigned to one of the groups. Assuming that the Htr clusters remain stable during the purification procedure, the different interactions of the Htr groups indicate the presence of different receptor clusters in *Hbt.salinarum*.

In addition to their interactions, Table 2 lists the number of predicted transmembrane helices for each Htr (retreived from HaloLex, [11]), an indication of whether the respective Htr is a transmembrane or a cytosolic protein. All Htrs found in groups 1 and 2 are transmembrane proteins, whereas the Htrs in groups 3 and 4 are cytosolic. No mixed transmembrane/cytosolic group was detected, which supports the hypothesis that Htrs from different groups belong to different receptor clusters.

The lack of detectable CheW binding to the Htrs from group 2 demonstrates that in *Hbt.salinarum* CheA can interact with Htrs directly, and that this interaction is stable even if no CheW protein is (stably) bound. For *E.coli*, there are contradictory results on the dependence of the receptor-CheA interaction on CheW. An early *in vitro* study suggests that CheW is necessary as adapter to mediate binding of CheA to receptors [19]. In contrast, a more recent study found that CheA could bind to the receptors independent of CheW and that CheW only strengthened the interaction [86]. An *in vivo* localization study found that truncated CheA constructs could bind to receptor clusters independently of CheW, whereas full-length CheA required CheW for this [87].

Only Htr group 1 matches the expected composition of prokaryotic taxis signaling complexes (receptor-transducer, CheW, CheA, CheY, [19,73]). Considering that binding of a CheW domain protein is mandatory for CheA activity [88-93], our findings indicate that only the receptors from group 1 were active under the tested conditions. At least for Htr11 (Car, the **c**ytoplasmic **a**rginine **r**eceptor, [42]), the only receptor with known signal that was assigned to a group other than group 1, this would make sense. *Hbt.salinarum* degrades arginine to ornithine coupled with the production of ATP [94]. This substrate-level phosphorylation allows the cells to grow in the absence of light and oxygen, making taxis towards arginine crucial under these conditions. Under the aerobic conditions used in our experiments, the cells can produce energy by oxidative phosphorylation. Arginine is indeed metabolized under aerobic conditions and is depleted rapidly from the medium, but it can be resynthesized from ornithine [95]. Consequently, the cells have no need for arginine uptake and arginine taxis could be switched off.

### Two novel interactors of CheA

**Figure 5:**
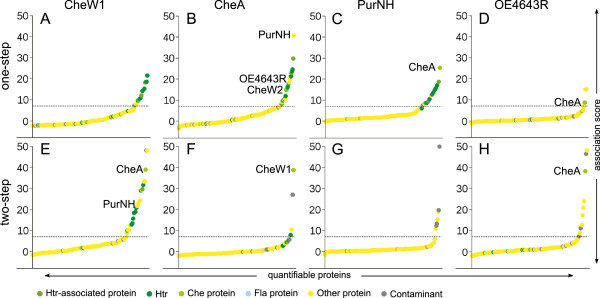
**Interactions of the core signaling proteins CheW1 and CheA and their novel interaction partners PurNH and OE4643R.** Plots show the association score of the proteins identified in one-step (A-D) or two-step (E-H) bait fishing experiments with CheW1 (A, E), CheA (B, F), PurNH (C, G) and OE4643R (D, H). The dashed line indicates the threshold used in this study for assuming an interaction. The proteins CheA, CheW1, CheW2, PurNH and OE4643R are labeled in the plots when identified with an association score above the threshold. For the underlying data see Additional file 3 and Additional file 4.

Two proteins were identified as novel interaction partners of CheA (Figures 3 and 5). The first is PurNH (OE1620R) which is annotated as a phosphoribosylglycinamide formyltransferase (EC 2.1.2.2) / phosphoribosylaminoimidazolecarboxamide formyltransferase (EC 2.1.2.3). Thus it carries out two essential enzymatic activities in purine metabolism. PurNH was fished by CheA, CheW1 and CheY (Figure 5). When PurNH was subsequently used as bait, it fished CheA and most of the group 1 Htrs. In all experiments, PurNH showed an interaction and exchange behavior identical to that of CheA (Additional file 4), indicating that it is statically bound to CheA.

The second novel interactor is OE4643R, a conserved protein of unknown function. OE4643R belongs to the uncharacterized protein family DUF151 (Pfam, [96]) and the cluster of orthologous groups COG1259 (“uncharacterized conserved protein”) [97,98]. A homologue of this protein from *Thermotoga maritima*, TM0160, has been crystallized and the structure solved to 1.9 Å resolution, but the function remains unclear [99].

Unlike PurNH, OE4643R was only fished with CheA and not with CheW1 and CheY (Figure 5, Additional file 4). When used as bait, OE4643R fished CheA but it did not reveal the typical association pattern of the core signaling proteins since neither CheW1 and nor Htrs with their associated proteins were copurified (Figure 5D, H). Hence OE4643R interacted with a pool of CheA not bound to Htrs.

In enterobacteria, two species of the CheA protein exist: *CheA*_*L*_, the full length protein, and *CheA*_*S*_, an N-terminally truncated form, which has an alternative translation initiation site [100]. In our experiments, the N-terminal peptide sequence of the Htr-bound pool of CheA (fished with CheW1) and the cytosolic pool (fished with OE4643R) were identical (Additional file 8). Thus N-terminal truncation is not the reason for the two pools of *Hbt.salinarum* CheA. Possibly, binding of CheA to OE4643R competes with its binding to Htrs and CheW1.

*Hbt.salinarum* CheA has the same domain composition as CheA from other organisms; no additional domain is present (data not shown). Thus the interactions with PurNH and OE4643R occur at common CheA domains, suggesting the possibility that similar interactions could take place in other organisms as well. However, we are not aware of any study reporting this and the functional role of the interactions of PurNH and OE4643R with the core signaling complex or CheA, respectively, remains unknown. Deletion of OE4643R or PurNH did not result in apparent chemotaxis defects in swarm plate assays (data not shown), indicating that these proteins have no essential function in the taxis signaling network but rather a regulatory function. Alternatively, OE4643R and PurNH could be part of yet unknown taxis signaling pathways that target CheA, similar to taxis signaling through PEP-dependent carbohydrate:phosphotransferase systems in bacteria [101].

### Only CheW1 is engaged in signaling complexes with CheA

Albeit quite widespread in bacteria [102] and archaea [10], the relevance of having more than one CheW protein in a chemotaxis signaling system is not clear. In our experiments, the two *Hbt.salinarum* CheW proteins showed different interactions with the Htrs and CheA. Both CheW proteins fished the group 1 and 3 Htrs. Whereas in one-step bait fishing with CheW2 the SILAC ratios of the Htrs equilibrated to one, they remained stable with CheW1. This indicates that the binding of CheW2 to the Htrs is more dynamic than the binding of CheW1. The difference in the affinity for CheA was much more apparent. In contrast to CheW1, which copurified with large amounts of CheA, CheW2 did not fish CheA at all. With CheA as the bait CheW2 was found as the prey in one-step bait fishing. However, this could also be due to copurification with assemblies of Htrs and does not necessarily indicate a direct interaction of CheW2 with CheA.

**Figure 6:**
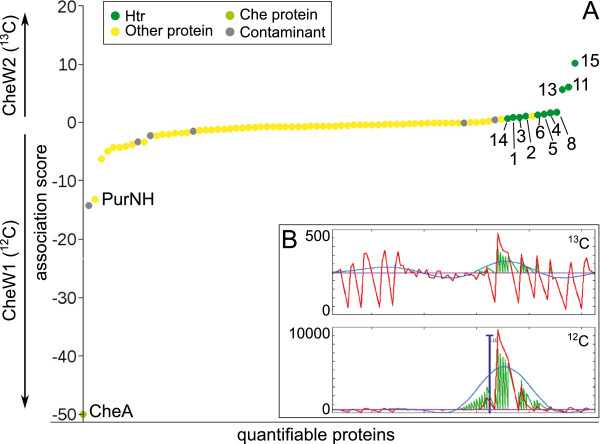
**Comparative bait fishing shows different interactions of the two CheW proteins.A** Plot of the association score of proteins identified in a comparative bait fishing experiment with both CheW proteins. Proteins bound to a higher extent to CheW2 than to CheW1 appear with a positive association score and proteins bound to higher extent to CheW1 than to CheW2 with a negative association score. Proteins bound to both baits to the same extent as well as background proteins appear with an association score close to 0. **B** Representative extracted ion chromatograms of a peptide of CheA (N-terminal peptide MDDYLEAFVR). The upper panel shows the ^13^C form (fished by CheW2) and the lower panel the ^12^C form (fished by CheW1).

To further study the roles of the two CheW proteins, a comparative bait fishing experiment was done (Figure 6). This experiment was performed as two-step bait fishing in which the second CheW was used as the control instead of plain CBD. CheW1 was bound to one cellulose column and incubated with light (^12^C) cell lysate. CheW2 was bound to a second column and incubated with heavy (^13^C) cell lysate. In this experiment, the light forms (^12^C) of CheA and PurNH were present in high amounts whereas the heavy forms (^13^C) were hardly detectable (see Figure 6B for representative chromatograms of a CheA peptide). This demonstrates strong binding to CheW1 and no or only weak binding to CheW2. The membrane-bound Htrs identified in this experiment (Htr1, 2, 3, 4, 5, 6, 8, 14; i. e. all Htrs from group 1) exhibited a SILAC ratio of nearly one, meaning they were bound to both CheWs to the same extent. The three cytoplasmic transducers Htr11 (Car), Htr13 and Htr15 (group 3) were purified to a higher extent with CheW2 than with CheW1.

These results are in perfect agreement with the single bait fishing experiments and show the following: (1) both CheW proteins have a similar affinity to accessible group 1 Htrs when added exogenously. CheW2 has a higher affinity to group 3 Htrs under these conditions; (2) CheW2 does not or only weakly binds CheA and forms complexes with Htrs to which CheA is not or only weakly bound; and (3) thus, under the tested conditions, only CheW1 is engaged in stable signaling complexes with CheA and Htrs.

A possible interpretation is that CheW2 competes with CheW1 for binding to the Htrs and thereby impedes the formation of signaling complexes. Hence CheW2 in *Hbt.salinarum* could play a role similar to that of CheV in *B.subtilis*, which contains a CheW-like domain and a response regulator domain [103] and disrupts functional receptor-CheA coupling [48]. This could happen on a fast time scale in response to CheA activity, which would then be an adaptation system like CheV [48]. Yet it seems more likely that CheW2, which unlike CheV has no response regulator domain, acts on a slower time scale, probably to tune the signaling impact of certain receptors according to the current demands of the cell as discussed above. This hypothesis is supported by the finding that the group 3 Htrs, where CheW2 binding exceeded CheW1 binding, were not fished by CheA. A similar effect could also be achieved when the interaction of CheA with the CheW proteins were regulated, i. e. if CheA develops a higher affinity for CheW2 under different growth conditions. By this, CheA could be recruited to the currently required Htrs, which could for example be group 3 Htrs under anaerobic growth conditions.

Another possible explanation is that CheW2 is the connection to an additional, not yet elucidated part of the taxis signaling system. The fumarate switch factor [49,50] could be a candidate here.

### Different protein complexes around the core signaling proteins and evidence for dynamic changes

AP-MS experiments inherently give only limited information about protein complex topology. However, the use of two complementary methods in this study made it possible to draw conclusions about the properties of the interactions in the core signaling complex.

**Figure 7:**
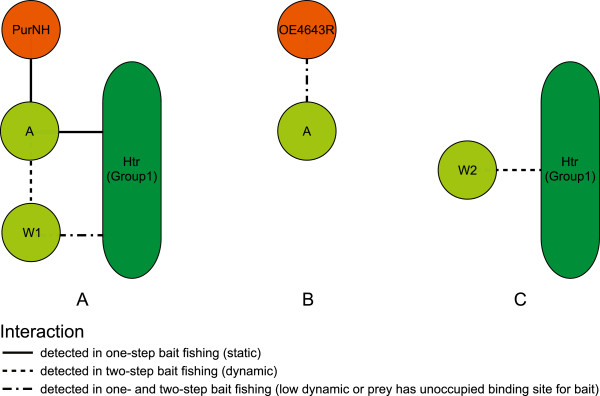
**Complexes of the core signaling proteins.** Different complexes in which the core signaling proteins are involved were reconstructed from the copurification data (see text). Colors and labels are as in Figure 3. Exchange rates between the different complexes cannot be deduced from our data. **A** Complex from Htrs, CheA, CheW1 and PurNH. Both CheA and CheW1 interact directly with the Htrs; PurNH interacts only with CheA. The interaction between CheA and CheW1 and possibly between CheW1 and the Htrs is dynamic. **B** A complex between CheA and OE4643R is not bound to Htrs or CheW1. **C** A complex of Htrs and CheW2 lacks CheA.

Additional file 9 shows results that were extracted from the complete results set (Additional file 3) which could lead to conclusions about the topology and properties of the core signaling protein complexes. The existence of three different protein complexes can be deduced from the data (Figure 7). (A) A complex between Htrs (group 1), CheA, CheW1 and PurNH. The interactions CheA-PurNH and CheA-Htr are static (deduced from observations 2, 3, 6, 7, 27, 28, 29 in Additional file 9). The interaction between CheA and CheW1 is dynamic (1, 5, 9, 12). The interaction CheW1-Htr was identified in one-step and two-step bait fishing (11, 14). This can be explained by either limited exchange of CheW1 in complexes containing Htrs, CheA and PurNH or by the presence of complexes containing Htrs, CheA and PurNH with free CheW1 binding sites. (B) A complex between CheA and OE4643R (4, 19, 23) which is not associated with CheW1 and Htrs (20-22, 24-26). The interaction CheA-OE4643R is either low dynamic or CheA which is accessible to exogenously added OE4643R is present in the cell (19, 23). The second alternative is more likely because OE4643R did not copurify in two-step bait fishing with CheA (8), which would be expected if the interaction were low dynamic. (C) A complex between CheW2 and Htrs (group 1) (15, 17) lacking CheA (16, 18). This interaction is dynamic (15, 17).

The dynamics in the CheA-CheW1 interaction as well as in the CheW1-Htr and CheW2-Htr interactions suggest that CheW binding to signaling complexes in *Hbt.salinarum* can undergo dynamic changes. Dynamic changes in the signaling clusters have recently been directly observed in *B.subtilis*[81]. Immunofluorescence microscopy showed that attractant binding caused a decrease in the number of observable polar receptor clusters and an increase in the lateral receptor clusters. The disappearance or appearance of receptor clusters is probably caused by an altered degree of receptor packing [81]. At the same time, the localization of CheV changed from primarily lateral to primarily polar. In striking similarity to our findings, the changes in CheV localization either require free binding sites or exchange between CheV and CheW at the polar receptor clusters. Thus, in *B.subtilis* the interactions of the CheW domain protein CheV, and possibly that of CheW, also exhibit dynamic changes.

Erbse and Falke found that the ternary signaling complexes of CheA, CheW and a chemotaxis receptor from *E.coli* or *Salmonella typhimurium* are “ultrastable” [104]. They demonstrated that CheA in the assembled complex does not exchange with its unbound form, even if added to the medium in 100-fold excess. This results are in perfect agreement with our observations. A similar experiment showed stable activity of the signaling complexes after addition of excess CheW; this suggests also static CheW binding. However, in our view these data do not strictly exclude exchange of CheW in the assembled signaling complex.

In contrast to our results in *Hbt. salinarum*, Schulmeister *et al.* determined an *in vivo* exchange time of about 12 min for both CheA and CheW in *E. coli* chemoreceptor clusters [61]. An explanation for this discrepancy could be different binding characteristics of CheW in *E. coli* on the one hand and *Hbt. salinarum* and possibly *B. subtilis* on the other. *E. coli* has neither multiple species of CheW nor CheV and thus possibly has no need for dynamics (i. e., fast kinetics) in CheW binding.

Overall many questions regarding the properties of core signaling complexes in *Hbt.salinarum* remain unanswered. Nonetheless, our findings demonstrate the presence of different complexes around the core signaling proteins and provide substantial evidence that the signaling complex is not a static assembly but displays considerable dynamics at the site of the CheW proteins.

We propose the following interpretation of the novel findings for the core signaling structure. The Htr groups reflect different receptor clusters. The signaling impact of the clusters can be tuned separately, which is manifested as dissimilar binding patterns of CheA, CheW1, CheW2 and CheY. One regulator of signaling impact might be CheW2, which competes with CheW1 either for binding to Htrs or to CheA in a adjustable manner. It thereby influences CheA recruitment to certain receptor clusters and thus the formation of specific signaling complexes. Finally, this allows *Hbt.salinarum* to adjust the impact of certain Htrs on the integrated taxis signal to its current demands. To test this hypothesis, we suggest modifying the expression levels of the CheW proteins. Due to the proposed competition of the CheW proteins, an increased CheW2/CheW1 ratio should (under aerobic conditions as used in this study) lead to decreased CheA activation by the group 1 Htrs.

### Different interactions indicate different roles of the three CheC proteins

Proteins of the CheC family are CheY-P phosphatases [28,105]. An interaction between CheC and CheD has been demonstrated in *B.subtilis*, *P.horikoshii* and *T.maritima*[29,32,66]. The genome of *Hbt.salinarum* codes for three CheC proteins [5,6].

The following interactions of the CheC proteins were detected: (1) CheC1 and CheC2 interact with each other. CheC3 did not interact with another CheC; (2) CheC2 and CheC3 interact with CheD; (3) CheC1 interacts with CheB; and (4) CheC2 interacts with the archaeal chemotaxis proteins CheF1 and CheF2, which in turn interact with the response regulator CheY.

It is noteworthy that CheC1 and CheC2, which interact with each other, both consist of only a single CheC domain, while CheC3, which did not interact with another CheC protein, consists of two CheC domains. This might indicate the presence of two functional CheC units in *Hbt.salinarum*, which both interact with CheD. However, since neither CheC2-CheB nor CheC1-CheF1/2 and CheC1-CheD interactions were detected, the CheC1-CheC2 interaction seems to be rather unstable, which argues against the formation of stable heterodimers between these proteins.

As mentioned above, our study showed that CheC1 interacted with CheB. The receptor methylesterase CheB is a key player in adaptation [89,106]. Its activity is controlled by the phosphorylation status of its response regulator domain [107,108]. Because its response regulator domain is homologous to that of CheY [109], it might be that CheC1 dephosphorylates the response regulator domain of CheB and thereby regulates CheB activity.

The interaction of CheC2 with CheF1 and CheF2, which both act at the interface between the Che system and the archaeal flagellum [10], might be analogous to *B.subtilis*, where the main CheY-P phosphatase, FliY, is located at the flagellar motor switch [28,110,111]. Although a direct interaction between CheY and CheC was not detected by our methods, our data provides evidence for CheY-P dephosphorylation at the flagellar motor switch in *Hbt.salinarum*. This is particularly noteworthy since phosphatase localization was found to be a conserved and important principle in bacterial chemotaxis systems [112].

### CheD has a central role in the Che protein interaction network

CheD is a highly conserved protein found in all chemotactic archaea [10] and most chemotactic bacteria [3,31]. CheD is a receptor deamidase in the bacteria *B.subtilis* and *T.maritima*[31,32]. Receptor methylesterase activity has also been ascribed to CheD in *T.maritima*[32]. Similar to the situation in *E.coli*[26,106], receptor deamidase and methylesterase activities have been detected in *Hbt.salinarum* CheB [25]. It is not clear whether both CheB and CheD deamidate and/or demethylate receptors in the latter organism [25]. Thus the function of the CheD protein in *Hbt.salinarum* remains to be elucidated.

We identified interactions between CheD and CheC2, CheC3, CheB, as well as CheF1, CheF2 and OE2401F. Hence CheD is a hub in the *Hbt.salinarum* Che protein interaction network. The high conservation of CheD among chemotactic bacteria and archaea [3] and the severe phenotype of a CheD deletion (almost complete loss of tactic capabilities; our unpublished results) support the hypothesis that this protein has a central role in the taxis signaling network. Of the interactions detected here, only CheC-CheD has been described before [29,66]. In *B.subtilis* an interaction of CheD with the MCPs was identified through Y2H analysis [113]. This interaction was not detected in the present study. This might be due to different functions of CheD in the two organisms. However, it seems more likely that the affinity of a putatively dynamic CheD-Htr interaction was simply not high enough for detection by our bait fishing methods.

### A CheD-dependent adaptation system in Hbt. salinarum?

The interactors CheC and CheD in *B.subtilis* form a feedback loop from CheY-P to the transducers and thereby constitute one of the three adaptation systems of this organism (the other two being the methylation/demethylation system of CheR and CheB, and the CheV system) [48]. CheC binding to CheD decreases the latter’s receptor deamidase activity [30]. Additionally and more important for adaptation, CheD regulates the activity of CheA [113]. CheY-P stabilizes the CheC-CheD complex, which in turn reduces CheA stimulation and thus closes the feedback circuit. Indeed, the CheY-P binding ability of CheC seems to be more important for *B.subtilis* chemotaxis than its enzymatic activity [30].

In contrast to *B.subtilis*, a direct regulation of CheA activity by CheD seems questionable in *Hbt.salinarum* since receptor deamidase or methylesterase activity in *Hbt.salinarum* have till now only been demonstrated for CheB and not for CheD [25]. Additionally, in *Hbt.salinarum* a CheY-dependent or CheY-P-dependent regulation of transducer demethylation was experimentally demonstrated by Perazzona and Spudich [114], which implies the presence of a slightly different adaptational mechanism. A predictive computational model of transducer methylation [47] strongly supports the possibility that in *Hbt.salinarum* CheY and not CheY-P is indeed the feedback regulator.

Based on these findings we used the detected interactions to propose an alternative feedback mechanism from the response regulator to the Htrs that might contribute to adaptation. The effector part of this hypothetical feedback loop would be CheB, which influences CheA activity through receptor demethylation and deamidation. As suggested by the detected interactions, CheB could be regulated by CheD and/or by CheC1. In analogy to the *B.subtilis* CheC, the receptor part of the feedback circuit would be CheC2 and/or CheC3 which sense either CheY-P or, more likely, CheY. These ”receptors” interact with the control center CheD and with CheC1 in the case of CheC2. Finally, the receptor demethylation and/or deamidation activities of CheB would respond to changes in CheY-P or CheY levels and thus regulate CheA activity. If CheD itself also acts as effector in *Hbt.salinarum* (by receptor deamidation and/or CheA regulation) remains to be investigated.

## Conclusions

In this study we analyzed the protein interaction network of an archaeal taxis signaling system. For the core signaling structure, the interaction analysis revealed: (1) the Htrs can be assigned to different groups according to their interactions with the core signaling proteins; (2) under the tested conditions, only CheW1 is engaged in signaling complexes with Htrs and CheA, whereas CheW2 builds complexes with Htrs but without CheA; and (3) the core signaling proteins are involved in different protein complexes and we have evidence for dynamic changes. Together, these findings indicate that basic properties of the archaeal core signaling structure are still not understood, possibly because they are not present in the best-studied taxis signaling system, the streamlined system of *E.coli*. We propose that *Hbt.salinarum* has the capability to selectively adjust the impact of certain Htrs or Htr clusters depending on its current needs or environmental conditions.

For the other Che proteins, our results show: (1) different interactions of the three CheC proteins indicating different functional roles; (2) a central role in the Che protein interaction network for CheD; and (3) interactions of CheB with CheC1 and with CheD. On the basis of these interactions we hypothesize that the CheCs, CheD and CheB build a feedback loop from the response regulator to Htr methylation.

Follow-up experiments are needed to assess the biological relevance of the interactions detected in this study and to test the hypotheses derived from the interactions. It will be interesting to see if the here described findings are restricted to archaeal taxis signaling systems or if they occur in bacterial systems as well.

## Methods

### Materials

Unless indicated otherwise all chemicals were purchased from Sigma-Aldrich (St. Louis, MO, USA), Merck (Darmstadt, Germany), or Fluka (Buchs, Switzerland) at the highest purity grade available. Restriction enzymes were purchased from New England Biolabs (Frankfurt, Germany). U-^13^C_6_-leucine was from Cambridge Isotope Laboratories (MA, USA).

### Strains and growth conditions

*Hbt.salinarum* strain R1 (DSM 671) was grown aerobically in the dark either in complex medium or in synthetic medium as described previously [115,116]. Transformation of *Hbt.salinarum* was performed essentially as described by [117]. Transformed cells were grown with 0*.*15 *μgml*^−1^ novobiocin (Sigma). *E.coli* strains DH5*α*, *ccdB* survival™2 *T*1^*R*^, Mach1™-*T*1^*R*^ and transformants were grown in LB medium (1% tryptone, 0.5% yeast extract, and 1% NaCl) at 37°C and supplemented with ampicillin (100 *μgml*^−1^), kanamycin (25 *μgml*^−1^), or chloramphenicol (50 *μgml*^−1^), if necessary.

### Construction of vectors

The plasmid pMS4 was obtained by cloning the promoter PrR16 [118,119] and the CBD (both amplified from the plasmid pWL-CBD [55] by PCR), the Gateway vector conversion cassette (Invitrogen), again the CBD, a His tag and transcriptional terminator from the *Hbt.salinarumbop* gene into the plasmid pVT [120] which provides a novobiocin resistance gene [121] and the *bgaH* marker gene [122] as well as an *E.coli* origin of replication and an ampicillin resistance cassette. pMS6 was derived from pMS4 by removing both CBDs by restriction digest with NcoI and XbaI and subsequent reconstitution of the Gateway cassette.

Gateway destination vectors were propagated in *ccdB* survival cells grown in LB medium containing chloramphenicol and ampicillin.

For generation of expression plasmids, bait protein coding sequences were amplified by PCR using the primers listed in Additional file 10 with Phusion polymerase (Finnzymes) according to supplier’s recommendations. The purified PCR products were cloned into the pENTR/D-TOPO vector (Invitrogen) according to manufacturer’s instructions, and transformed into *E.coli* One Shot®;Mach1™-*T*1^*R*^competent cells. Kanamycin-resistant (kanR) colonies were screened by colony PCR using the primers M13F (-20) and M13R (-26) to verify insert size, and positive clones sequence-verified using the same primers. Inserts were shuttled into pMS4 and pMS6 using Gateway®;LR Clonase™II Enzyme mix (Invitrogen) and the resulting expression plasmids verified by restriction digest.

### Generation of *Hbt.salinarum* bait expression strains

Expression plasmids were transformed into *Hbt. salinarum* R1. Transformants were identified by their novobiocin resistance and their blue color on X-gal containing plates. Expression of the tagged bait protein in pMS4 transformants was verified by affinity purification on cellulose and subsequent PAGE. Bait-control strains transformed with pMS6 were checked by western blot with an anti-penta-his HRP conjugate (QIAGEN).

### Affinity purification of CBD-tagged proteins

The bait expression strain was precultured in 35 ml complex medium containing 0*.*15 *μgml*^−1^ novobiocin at 37°C on a shaker (150 rpm) until an *OD*_600_of 0.6 was reached. This preculture was used to inoculate 100 ml complex medium at an *OD*_600_ of 0.01. When the main culture had reached an *OD*_600_of 0.6 to 1.0, cells were harvested by centrifugation (8000 rpm, 15 min, 15°C) and resuspended in 1-2 ml CFE buffer (3 M KCl, 1 M NaCl, 400 mM *NH*_4_*Cl*, 40 mM *MgCl*_2_, 10 mM Tris/HCl, pH 7.5) plus protease inhibitor (Complete Mini, EDTA-free, Roche) (CFE + PI). Cells were lysed by sonication on ice water (2 × 20 sec, Branson sonifier 250, 3 mm disruptor horn, output level 2, constant), and the lysate cleared by centrifugation at 14000 rpm, 18°C for 20 min in a tabletop centrifuge.

A cellulose column was prepared by pipetting 30 mg Avicell PH-101 (Fluka) resuspended in 300 *μl*CFE into a Mobicol empty spin column (MoBiTec). The column was centrifuged (300 × g, 1 min, RT), washed with 600 *μl*CFE to remove fines and centrifuged again.

The cleared lysate was applied to the column in 600 *μl*portions and the cellulose resuspended by pipetting up and down. After 1 min incubation at room temperature, the column was centrifuged (300 × g, 1 min, RT) and the flow-through discarded. The cellulose was washed three times with 600 *μl*CFE + 0.5% NP40 (Roche) and once with CFE. After each washing step the column was centrifuged (300 × g, 1 min, RT) and the flow-through discarded. An additional centrifugation (770 × g, 1 min, RT) was performed after the last washing step to reduce the amount of retained buffer. For elution, 600 *μl* ethylene glycol (Merck, Darmstadt) were applied to the column, the cellulose resuspended, and the column centrifuged. Eluted proteins were precipitated with TCA. For this, an equal volume of 20% (w/v) TCA was added to the eluate, the mixture incubated on ice for 30 min and centrifuged at 14000 rpm, 4°C, 30 min. Finally, the pellet was washed 2-3 times with ice-cold 50% (w/v) acetone.

For SILAC-based one-step bait-fishing experiments the above protocol was modified as follows:

The bait expression strain and the bait-control strain were precultured in 35 ml complex medium containing 0*.*15 *μgml*^−1^ novobiocin at 37°C on a shaker (150 rpm) until an *OD*_600_of 0.5-1.0 was reached. Five hundred microliters of these cultures were used to inoculate second precultures that were grown under identical conditions to an *OD*_600_of 0.8-1.0. The second precultures were used to inoculate 100 ml synthetic medium containing ^13^C_6_-leucine for the bait expression strain and ^12^C_6_-leucine for the bait-control strain at an *OD*_600_ of 0.01; the inoculum was adjusted to 1.5 ml with complex medium before addition to the 100 ml medium.

The main cultures were incubated on a shaker (110 rpm) at 37°C in the dark until they reached an *OD*_600_ of 0.8. Cells were harvested by centrifugation (8000 rpm, 15°C, 15 min) and pellets resuspended in 1 ml CFE + PI. Cell lysate and cellulose columns were prepared as described above. Three hundred microliters lysate from each culture were applied to the column, the cellulose resuspended, and after 1 min incubation the column centrifuged (300 × g, 1 min, RT). This step was repeated twice, followed by washing, elution, and protein precipitation as described.

Two-Step bait-fishing experiments were performed with the following modifications:

*Hbt.salinarum* R1 was precultured twice in 35 ml complex medium at 37°C on a shaker (110 rpm) until an *OD*_600_ of 0.5-1.0 was reached. When the second preculture had reached an *OD*_600_of 0.8-1.0, it was used to inoculate two cultures with 100 ml synthetic medium containing either ^13^C_6_-leucine or ^12^C_6_-leucine at an *OD*_600_of 0.01. The inoculum was brought to a total volume of 1.5 ml with complex medium. The cultures were incubated on a shaker (110 rpm) at 37°C in the dark until they had reached an *OD*_600_ of 0.8.

In parallel, the bait expression strain and the CBD-control strain were precultured as described before. When an *OD*_600_of 0.8-1.0 was reached 200 ml complex medium were inoculated at an *OD*_600_of 0.01 and incubated at 37°C on a shaker (110 rpm). The main cultures were harvested at an *OD*_600_ of around 1.0. Cells of all four cultures were pelleted and lysed and two cellulose columns were prepared as described above. Six hundred microliters lysate from the bait expression culture or the CBD-control culture were applied to each cellulose column, the cellulose resuspended and after 1 min incubation, the columns centrifuged (300 × g, 1 min, RT). This step was repeated, and the columns washed three times with 600 *μl* CFE + 1% NP40 + 20% ethylene glycol and once with CFE.

Lysate from the *Hbt.salinarum* R1 wt cells was applied to the columns in 600 *μl*portions (cells labeled with ^12^C_6_-Leucine for the bait column and with ^13^C_6_-Leucine for the CBD-control column), the cellulose resuspended and after 1 min incubation, the column centrifuged (300 × g, 1 min, RT). Washing and elution were done as described above. The eluates from both columns were pooled and proteins precipitated as described.

### Mass spectrometry

Precipitated proteins were separated on 4-12% Bis Tris gels (NuPAGE, Invitrogen) and stained with Coomassie Brilliant Blue R250. For LC-MS/MS analysis, the entire lane was removed from the gel and divided into 10-15 slices. The size of the slices was chosen according to the estimated number of tryptic peptides derived from the respective part of the lane. Additionally, very thick bands were separated from weaker ones to prevent masking of low-abundance proteins. Slices were cut into pieces of circa 1 *mm*^3^. Digestion and elution were performed essentially as described by Shevchenko [123]. Peptides were desalted by reverse phase (RP) chromatography using self-packed Stage tips (STop And Go Extraction, [124]). Protein identification by nanoLC-MS/MS was done on a ESI Q-TOF Ultima mass spectrometer (Waters, Milford, MA) as described in [125] with minor modifications.

Briefly, the dried peptides were dissolved in 20 *μl*5% formic acid, and 1-6 *μl*(depending on the amount of protein estimated by the intensity of the Coomassie blue-stained gel) were loaded into the CapLC (Waters) using an auto sampler. They were bound to the precolumn (self-packed, 100 *μm*× 25 mm ReproSil-Pur 200 ^18^C-AQ, 5 *μm*, Dr. Maisch GmbH, Ammerbuch-Entringen, Germany) with a flow rate of 2 *μlmin*^−1^ and analyzed on the main column (self-packed, 75 *μm*×150 *mm *ReproSil-Pur 200 ^18^C-AQ, 3 *μm*) with a flow rate of 200 *nlmin*^−1^. Bound peptides were eluted in an linear acetonitrile gradient and injected into the mass spectrometer.

Mass spectrometric analysis was done in *positive ion mode* with a capillary voltage of 2.3 kV. The mass window was set to 300-2000 Da in MS mode and 50-2000 Da in MS/MS mode. Survey scans were acquired for 1.5 s. From each survey scan up to four peptides were chosen for fragmentation; selection criteria were the signal intensity and the charge state (at least two fold). CID was performed with a collision voltage between 16 and 40 kV and helium as collision gas.

### Data analysis

Peak lists were extracted from the raw data with Mascot Distiller (V. 2.3.1.0, Matrix Science Ltd., London, UK) and submitted to an in-house Mascot server (V. 2.2.06, Matrix Science) for searches against a *Halobacterium salinarum* R1 protein sequence database. Carbamidomethylation of cysteine was set as a required modification and oxidation of methionine and acetylation of the protein N-terminus as variable modifications. Up to three missed tryptic cleavage sites were allowed. For SILAC experiments, ^13^C_6_-Leucine was additionally set as variable modification. Mass tolerance was set to 1.5 Da for MS and 0.6 Da for MS/MS.

Protein ratios of SILAC experiments were determined with ASAPRatio [126] embedded in the Trans-Proteomic Pipeline (TPP)[127]. ASAPRatioPeptideParser was used with the options “lL” (set leucine as labeled residue), “C” (quantitate only the charge state where the CID was made), “B” (return a ratio even if the background is high), and “F” (use fixed scan range for light and heavy peptide). All other TPP tools were run with default parameters. Protein ratios were checked manually on basis of the extracted ion chromatograms and adjusted if necessary (e. g. background level or scan range). Only protein identifications with at least two identified peptides, a ProteinProphet probability [64] of 0.95 or higher and a valid protein ratio were accepted.

For a better presentability, of the protein ratios a symmetrical measure called association score, was introduced. The association score was calculated from the SILAC ratio (bait isotopic form divided by control isotopic form) as follows:

$$\text{AssociationScore}=\left\{\begin{array}{ll} \text{SILACRatio}-1 & \text{if}\text{SILACRatio}\geq 1\\ 1-\frac{1}{\text{SILACRatio}} & \text{if}\;\text{SILACRatio}<1 \end{array}\right)$$

To account for dynamic range limits of the QTOF mass spectrometer and facilitate graphical representation, the association score was limited to a maximum of 50. In cases of sticky baits, i. e., bait proteins which copurified with more than 20 proteins with an association score > 3, the association score was reduced by 2 for all identified proteins.

Prey proteins were considered to be interaction partners if they were identified with an association score > 7. Proteins that were identified as binders of the CBD in control experiments and proteins that appeared as interactors in almost all experiment were marked as ”contaminants” and removed from the final data set. These proteins are listed in Additional file 11. More details on the evaluation of the results from the bait fishing experiments are given in Additional file 2.

## Competing interests

The authors declare that they have no competing interests.

## Author’s contributions

MS, HB and DO conceived and designed the experiments. MS and HB established the bait fishing method and JM, AM and MS performed the bait fishing experiments. BS, MA and FS performed the mass spectrometric measurements, MS analyzed the MS data and performed the computational analysis. MS produced the figures and wrote the manuscript. HB, MA, FS and DO revised the manuscript. All authors read and approved the final manuscript.

## Supplementary Material

Additional file 1 **Expression of the CBD-tagged bait protein and the untagged control.A, B** Schematic representation of the bait-CBD expression vector pMS4 and the corresponding bait-control pMS6. Both plasmids contain a pUC origin (not indicated) and an ampicillin resistance (AmpR) for amplification in *E. coli*. The novobiocin resistance (NovR) and *β*-galactosidase (*bgaH*) are for selection of transformants in *Hbt. salinarum*. Bait genes are cloned between the *attR1* and *attR2* sites via Gateway recombination (Invitrogen). Between the bait protein and the CBDs (pMS4) or the His-Tags (pMS6) is a short linker sequence (IGAVEER, the linker of the two *β*-sheets in *Hbt. salinarum* dodecin). Downstream of the fusion protein is a transcriptional terminator from the *Hbt. salinarumbop* gene (not shown). **C, D** The plasmids do not contain a haloarchaeal origin of replication. After transformation into *Hbt. salinarum*, they are integrated into the genome at the site of the bait protein by homologous recombination. **C** Integration of pMS4 constructs (red) into the genome (blue) leads to the expression of the bait C-terminally fused to CBD under control of the bait’s endogenous promoter and the expression of an N-terminal bait-CBD fusion under control of the promoter PrR16 (a highly active, modified ferredoxin promoter [118,119]). **D** Integration of pMS6 constructs results in similar promoter-bait constructs without CBD. Click here for file

Additional file 2 Details on result evaluation of the bait fishing experiments. Click here for file

Additional file 3 Protein identifications in bait fishing experiments. Click here for file

Additional file 4 **Identification of the core signaling proteins in all bait fishing experiments.** The numbers show the sequence coverage of the protein identification. Numbers in bold type indicate that this protein was identified as an interaction partner by the SILAC ratio. Numbers in italics indicate that this prey was identified with relatively high sequence coverage in a one-step bait fishing experiment but the SILAC ratio was close to one and that this prey was identified as an interaction partner in two-step bait fishing. Together, this indicates a dynamic interaction between bait and prey. Click here for file

Additional file 5 **Bait fishing experiments for the Che interaction network.** The upper part of the table shows the initial experiments with the 10 *Hbt. salinarum* Che proteins known before the start of this study. The lower part lists experiments with baits which were identified as interaction partners in the initial experiments. Interaction analysis revealed that two of these (OE2402F and OE2404R) were novel, archaea-specific Che proteins [10]. Minus indicates that experiments were not included in the final dataset because of too many proteins were bound (more than 20 unexpected interactors with an association score > 7). ^*^ This experiment was not done with reversed isotopic labeling. Thus some putative interactors (found in the one-step experiment) have a negative association score. ^**^ One-Step bait fishing with CheB was repeated after weak bait protein binding in the first attempt. Results from both replicates were included into the final dataset. Click here for file

Additional file 6 Chemotaxis protein interaction network. Click here for file

Additional file 7 Physical and functional interactions in prokaryotic taxis signaling systems from literature. Click here for file

Additional file 8 **CheA peptides identified in bait fishing experiments with CheW1 and OE4643R give no indication for different CheA subspecies.** The complete CheA protein sequence is shown. Peptides in italics were identified with OE4643R and peptides shown underlined with CheW1. Click here for file

Additional file 9 **Observations characterizing protein complexes of the core signaling proteins.** Preys identified with relatively high sequence coverage but a SILAC ratio close to one in one-step bait fishing and identified as interactors in two-step bait fishing (Additional file 4) were assumed to exchange. For the underlying data see Additional file 3 and Additional file 4. Click here for file

Additional file 10 Primers used in this study. Click here for file

Additional file 11 Proteins considered to be contaminants. Click here for file
