# Supplementary figures and images for: The protein interaction network of a taxis signal transduction system in a Halophilic Archaeon
Source: BMC Microbiol. 2012 Nov 21;12:272. doi: 10.1186/1471-2180-12-272 (PMC3579733; doi:10.1186/1471-2180-12-272)

A

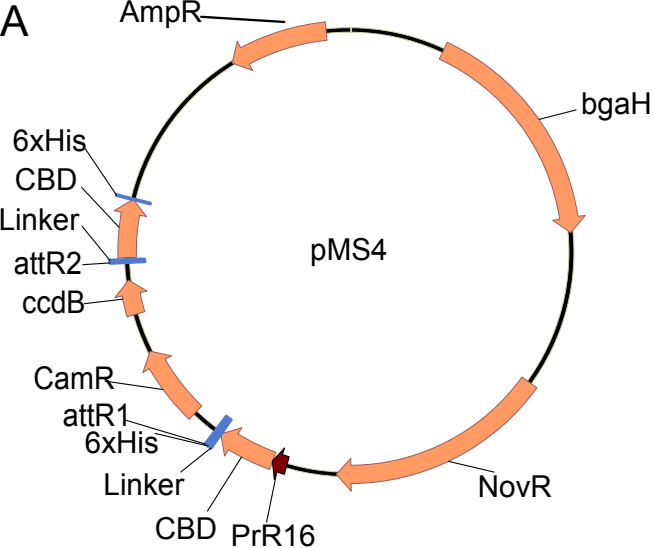

B

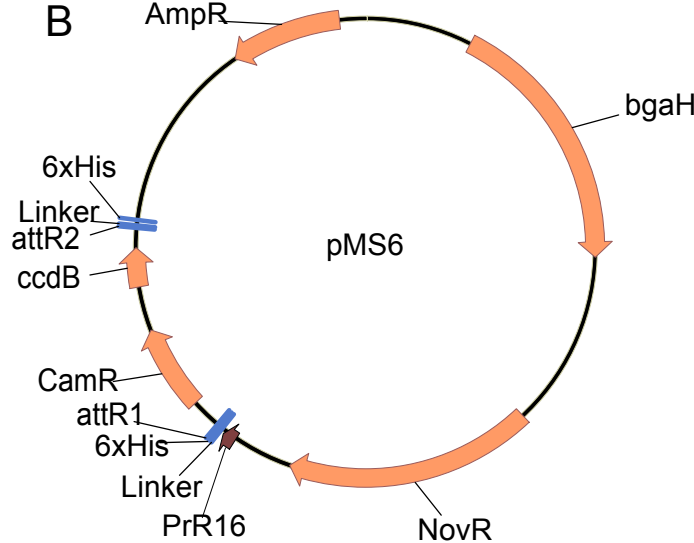

C

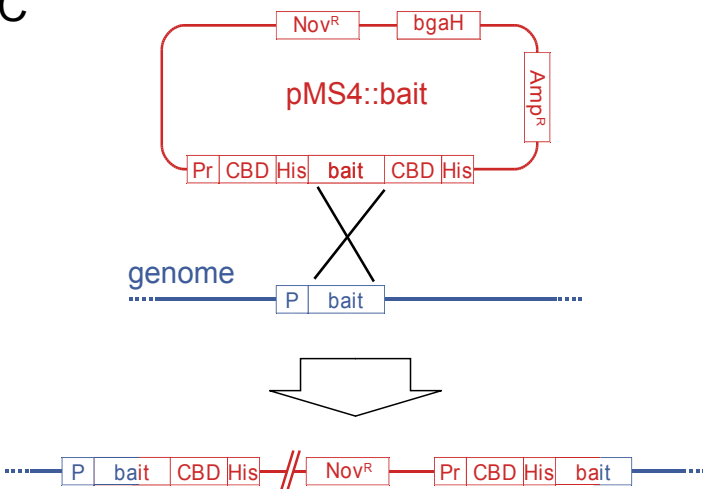

D

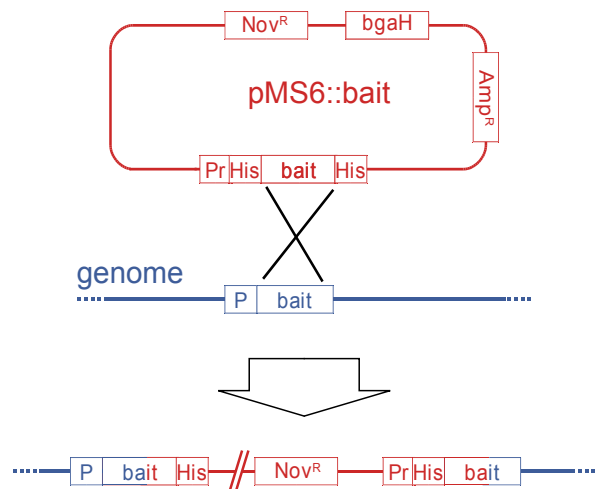

Supplement: Additional file 1 — Expression of the CBD-tagged bait protein and the untagged control.A, B Schematic representation of the bait-CBD expression vector pMS4 and the corresponding bait-control pMS6. Both plasmids contain a pUC origin (not indicated) and an ampicillin resistance (AmpR) for amplification in E. coli. The novobiocin resistance (NovR) and β-galactosidase (bgaH) are for selection of transformants in Hbt. salinarum. Bait genes are cloned between the attR1 and attR2 sites via Gateway recombination (Invitrogen). Between the bait protein and the CBDs (pMS4) or the His-Tags (pMS6) is a short linker sequence (IGAVEER, the linker of the two β-sheets in Hbt. salinarum dodecin). Downstream of the fusion protein is a transcriptional terminator from the Hbt. salinarumbop gene (not shown). C, D The plasmids do not contain a haloarchaeal origin of replication. After transformation into Hbt. salinarum, they are integrated into the genome at the site of the bait protein by homologous recombination. C Integration of pMS4 constructs (red) into the genome (blue) leads to the expression of the bait C-terminally fused to CBD under control of the bait’s endogenous promoter and the expression of an N-terminal bait-CBD fusion under control of the promoter PrR16 (a highly active, modified ferredoxin promoter [118,119]). D Integration of pMS6 constructs results in similar promoter-bait constructs without CBD. [file 1471-2180-12-272-S1.pdf]

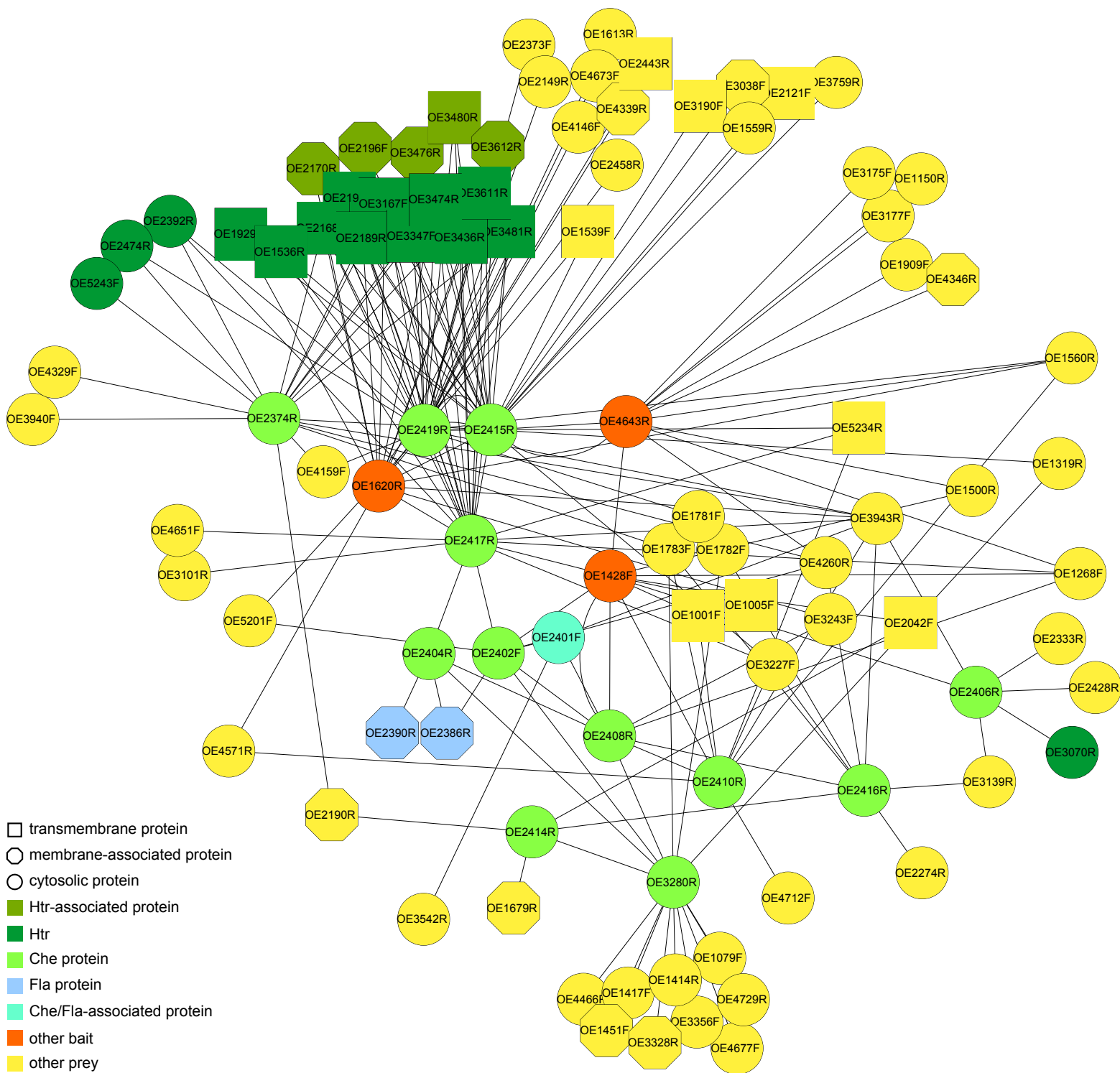

Supplement: Additional file 6 — Chemotaxis protein interaction network. [file 1471-2180-12-272-S6.pdf]
