# Supplementary material for: The protein interaction network of a taxis signal transduction system in a Halophilic Archaeon
Source: BMC Microbiol. 2012 Nov 21;12:272. doi: 10.1186/1471-2180-12-272 (PMC3579733; doi:10.1186/1471-2180-12-272)
Supplement: Additional file 2 — Details on result evaluation of the bait fishing experiments. [file 1471-2180-12-272-S2.pdf]

One-step and two-step bait fishing: Evaluation of results

Selection of reliable interactions

The evaluation of the SILAC AP-MS experiments required a threshold for the SILAC ratio to define when an identified protein should be considered a contaminant or putative interaction partner.

Other studies apply a statistical measure such as the Grubbs outlier test [1] or the z-test [2] to measure whether the deviation of a SILAC ratio is significant. These tests presuppose that the parameter to be tested follows a standard normal distribution in the test population (or at least in the fraction considered to be background). This prerequisite is not met for most datasets produced in this study (checked by Shapiro-Wilk normality test, not shown), making these tests inapplicable.

Hence, a fixed threshold for rating a prey protein as an interactor or contaminant was defined based on our observations. Manual inspection of the results revealed that in most experiments there was a clear separation of the vast majority of proteins with association scores of less than 3 from a few clearly enriched proteins with much higher association scores. For putative membrane-associated baits (e. g., proteins from the core signaling complex) and sticky baits, no clear separation between the majority of proteins and putative interactors was achieved at this asociation score level. Here the number of proteins first started to decline rapidly at an association score of around 5 to 7, indicating that higher association scores were required for membrane associated interactions. Based on these observations, the threshold for accepting an interaction was set at an association score of 7.

Without a doubt this fixed threshold neither represents the correct score for all cases nor can it measure the "quality of the guess" as in a probability-based score. However, the use of a rather conservative threshold of 7 should result in a relatively low false positive rate, albeit at the expense of an elevated false negative rate. Furthermore, almost all interactions discussed in this manuscript were identified with an association score of 10 or higher and thus are highly unlikely to be false positives.

Contaminants

To identify contaminants that bind to the CBD and therefore appear to be interactors, an one-step and a two-step bait fishing experiment were performed in which the CBD expression strain (MS4) was tested against wild type *Hbt. salinarum*. The proteins detected as "interactors" of CBD in these control experiments were designated contaminants. Furthermore, some other proteins were added to the background protein set because they either bound to the cellulose column when incubated with wild type *Hbt. salinarum* cell lysate (the chitinases OE2201F, OE2205F, and OE2206F) or they were highly promiscuous (i. e., found in almost every experiment) and were involved in protein folding or degradation. Since the bait is overexpressed and the CBD is from a non-halophilic organism, the bait might have been partially misfolded so that proteins involved in protein folding and degradation bound to it. The complete list of contaminants is shown in Additional File 11.

Reproducibility

Of the 201 interactions listed in Additional File 5, 23 were reproducible; which means they were detected in more than one experiment. This low number is due to the fact that most interactions had no chance of being identified more than once. All baits were actually used in two experiments (one-step and two-step bait fishing), but the different methods identify different interactions.

The interactions of only four protein pairs could be reciprocally confirmed (i. e., found in both bait-prey combinations: protein X was a prey of bait Y, and Y was a prey of bait X). There are several possible reasons for this. First, only some of the preys were later used as baits for reciprocal fishing. Hence for all others, a reciprocal confirmation per se was not possible. A second reason is steric hindrance by the relatively large CBD tag (molecular weight, 18 kDa) might prevent the formation of interactions in some

cases. Third, some proteins are more difficult to identify than others, so that certain preys were missed. Small proteins might be lost during the sample preparation procedure [3]. An example of this is the CheA-CheY interaction. When the small protein CheY (13.4 kDa) is used as bait, it is easy to identify CheA (71.8 kDa) as the prey. But when CheA was the bait CheY was not identified as the prey in any experiment. In addition, if a prey is located in the same gel slice as the bait it might be missed because its identification is hampered by the large amount of bait in the sample.

References

1. Selbach M, Mann M: **Protein interaction screening by quantitative immunoprecipitation combined with knockdown (QUICK).** *Nat Methods* 2006, **3**(12):981–983, [[http://dx.doi.org/10.1038/nmeth972]].

2. Dobreva I, Fielding A, Foster L, Dedhar S: **Mapping the Integrin-Linked Kinase Interactome Using SILAC.** *J Proteome Res* 2008, **7**(4):1740–1749, [[http://dx.doi.org/10.1021/pr700852r]].

3. Klein C, Aivaliotis M, Olsen JV, Falb M, Besir H, Scheffer B, Bisle B, Tebbe A, Konstantinidis K, Siedler F, Pfeiffer F, Mann M, Oesterhelt D: **The low molecular weight proteome of *Halobacterium salinarum*.** *J Proteome Res* 2007, **6**(4):1510–1518, [[http://dx.doi.org/10.1021/pr060634q]].
