# Supplementary material for: The protein interaction network of a taxis signal transduction system in a Halophilic Archaeon
Source: BMC Microbiol. 2012 Nov 21;12:272. doi: 10.1186/1471-2180-12-272 (PMC3579733; doi:10.1186/1471-2180-12-272)
Supplement: Additional file 4 — Identification of the core signaling proteins in all bait fishing experiments. The numbers show the sequence coverage of the protein identification. Numbers in bold type indicate that this protein was identified as an interaction partner by the SILAC ratio. Numbers in italics indicate that this prey was identified with relatively high sequence coverage in a one-step bait fishing experiment but the SILAC ratio was close to one and that this prey was identified as an interaction partner in two-step bait fishing. Together, this indicates a dynamic interaction between bait and prey. [file 1471-2180-12-272-S4.pdf]

| **Bait** | **Type** | **CheW1** | **CheA** | **PurNH** | **4643** | **Htr1** | **Htr2** | **Htr3** | **Htr4** | **Htr5** | **Htr6** | **Htr8** | **Htr14** |
|---|---|---|---|---|---|---|---|---|---|---|---|---|---|
| OE1428F | one-step | | 2.5 | | | | 6.2 | 4.3 | 10.8 | | 5.3 | 7.5 | |
| OE1428F | two-step | | | | | 34.1 | 5.0 | 21.5 | 15.7 | 23.6 | 15.6 | 8.7 | 5.9 |
| PurNH | one-step | 19.1 | **13.8** | | | 11.0 | **9.4** | **18.1** | **23.9** | **26.3** | **18.4** | **9.5** | |
| PurNH | two-step | | | | | | | | | | | | |
| CheW2 | one-step | 25.8 | 14.1 | 15.8 | | *51.3* | *12.4* | *29.8* | *29.2* | *37.9* | *26.0* | *18.4* | 7.0 |
| CheW2 | two-step | | | | | **20.5** | **6.0** | **7.7** | **10.8** | **20.0** | **11.9** | **7.5** | |
| OE2401F | one-step | 16.3 | | | | | | | 4.4 | | | | |
| OE2401F | two-step | | | | | | 7.2 | 14.8 | 14.9 | 20.2 | 11.0 | 7.5 | |
| OE2402F | one-step | | | | | | | | | | | | |
| OE2402F | two-step | | | | | | | | 2.3 | | | | |
| OE2404R | one-step | | | | | | | | | | | | |
| OE2404R | two-step | | | | | | | | | | 1.8 | | 2.2 |
| CheR | one-step | | 6.7 | | | 15.7 | 6.9 | 12.5 | 11.6 | 7.0 | 5.4 | 10.0 | |
| CheR | two-step | | | | | | | | | | | | |
| CheR | two-step | | 2.5 | | | | 6.0 | 5.2 | 12.5 | | 7.4 | 12.0 | 6.5 |
| CheD | one-step | 19.1 | | 18.6 | | 19.4 | 3.5 | | 10.9 | | | | |
| CheD | two-step | | | | | | 3.7 | | 5.3 | | | 5.0 | |
| CheC3 | one-step | | | | | 17.4 | 6.8 | 9.3 | 12.5 | 16.8 | 6.3 | 3.9 | |
| CheC3 | two-step | | | | | 11.8 | 7.1 | 16.5 | 14.0 | 27.3 | 20.5 | 9.8 | |
| CheC1 | one-step | | | | | | 2.4 | 7.0 | 7.8 | 8.0 | | | |
| CheC1 | two-step | | | | | 34.7 | 4.8 | 6.1 | 10.8 | | | 7.5 | |
| CheA | one-step | *43.8* | | **52.4** | **34.9** | **51.7** | **16.1** | **27.3** | **30.2** | **28.4** | **18.9** | **19.3** | **12.1** |
| CheA | two-step | **48.3** | | | | **49.1** | | | 16.3 | | 4.6 | 12.4 | |
| CheB | one-step | | | | | | 3.4 | | 7.8 | | | | |
| CheB | one-step | | 2.5 | | | | 6.2 | | 8.0 | | 3.9 | | |
| CheB | two-step | | | | | 11.2 | 3.7 | | 3.9 | | | | |
| CheY | one-step | 12.4 | *16.9* | | | *38.4* | *17.3* | *15.3* | *26.6* | *19.8* | *19.9* | *15.6* | *6.2* |
| CheY | two-step | | **20.1** | **9.9** | | **56.7** | **24.0** | **31.2** | **27.6** | **34.4** | **28.3** | **15.6** | **7.8** |
| CheW1 | one-step | | *37.0* | *35.1* | | **45.3** | **26.4** | **35.0** | **27.1** | **44.2** | **36.9** | **29.1** | **17.2** |
| CheW1 | two-step | | **32.5** | **19.3** | | **35.4** | **13.1** | **13.9** | **22.9** | **24.2** | **21.7** | **13.7** | **6.9** |
| CheC2 | one-step | | 2.5 | 10.4 | | | 3.7 | | 7.8 | | | | |
| CheC2 | two-step | | 8.5 | 5.9 | | 27.4 | 6.0 | 12.7 | 14.0 | 20.6 | 9.5 | 8.7 | |
| OE4643R | one-step | 19.1 | **23.1** | 14.1 | | 9.9 | | | 3.0 | | | 5.0 | |
| OE4643R | two-step | | **25.6** | 21.4 | | 21.1 | 4.8 | | 12.6 | | 10.5 | 9.6 | 4.8 |
