# Supplementary material for: The protein interaction network of a taxis signal transduction system in a Halophilic Archaeon
Source: BMC Microbiol. 2012 Nov 21;12:272. doi: 10.1186/1471-2180-12-272 (PMC3579733; doi:10.1186/1471-2180-12-272)
Supplement: Additional file 5 — Bait fishing experiments for the Che interaction network. The upper part of the table shows the initial experiments with the 10 Hbt. salinarum Che proteins known before the start of this study. The lower part lists experiments with baits which were identified as interaction partners in the initial experiments. Interaction analysis revealed that two of these (OE2402F and OE2404R) were novel, archaea-specific Che proteins [10]. Minus indicates that experiments were not included in the final dataset because of too many proteins were bound (more than 20 unexpected interactors with an association score > 7). * This experiment was not done with reversed isotopic labeling. Thus some putative interactors (found in the one-step experiment) have a negative association score. ** One-Step bait fishing with CheB was repeated after weak bait protein binding in the first attempt. Results from both replicates were included into the final dataset. [file 1471-2180-12-272-S5.pdf]

| **Bait** | | **Experiment** | |
|---|---|---|---|
| **Gene** | **Protein** | **One-step** | **Two-step** |
| OE2374R | CheW2 | + | +* |
| OE2406R | CheR | + | - |
| OE2408R | CheD | + | + |
| OE2410R | CheC3 | + | + |
| OE2414R | CheC1 | + | + |
| OE2415R | CheA | + | + |
| OE2416R | CheB | +** | + |
| OE2417R | CheY | + | + |
| OE2419R | CheW1 | + | + |
| OE3280R | CheC2 | + | + |
| OE1428F | | + | - |
| OE1620R | PurNH | + | + |
| OE2401F | | + | + |
| OE2402F | CheF1 | + | + |
| OE2404R | CheF2 | + | + |
| OE4643R | | + | + |
