# Supplementary material for: The protein interaction network of a taxis signal transduction system in a Halophilic Archaeon
Source: BMC Microbiol. 2012 Nov 21;12:272. doi: 10.1186/1471-2180-12-272 (PMC3579733; doi:10.1186/1471-2180-12-272)
Supplement: Additional file 7 — Physical and functional interactions in prokaryotic taxis signaling systems from literature. [file 1471-2180-12-272-S7.pdf]

Physical and functional interactions in prokaryotic taxis signaling systems from literature.

Interaction	Species and reference
CheA-CheB	*E. coli* [46, 133]
CheA-CheC	*B. subtilis* [96]
CheA-CheW	*C. jejuni* [44], *E. coli* [46, 48, 68, 134], *H. pylori* [43], *T. denticola* [135]
CheA-CheX	*T. denticola* [135]
CheA-CheY	*C. jejuni* [44], *E. coli* [46, 48, 133, 136–139], *H. pylori* [43], *T. denticola* [135], *T. maritima* [140]
CheA-CheZ	*E. coli* [46, 141, 142]
CheA-MCP	*E. coli* [61, 62]
CheB-MCP	*E. coli* [46]
CheC-CheD	*B. subtilis* [84], *P. horikoshii* [40], *T. maritima* [85]
CheC-MCP	*B. subtilis* [96]
CheD-MCP	*B. subtilis* [96], *T. maritima* [85]
CheR-MCP	*E. coli* [46], *S. typhimurium* [143]
CheV-MCP	*C. jejuni* [44]
CheW-MCP	*C. jejuni* [44], *E. coli* [46, 48, 68], *H. pylori* [43], *T. denticola* [135],
CheY-FliM	*E. coli* [46, 59, 144]
CheY-CheZ	*E. coli* [46, 51, 145, 146]
CheA phosphorylates CheB	*B. subtilis* [147], *E. coli* [117, 118]
CheA phosphorylates CheV	*B. subtilis* [147]
CheA phosphorylates CheY	*B. subtilis* [119], *E. coli* [117], *Hbt. salinarum* [116]
CheB deamidates MCP	*E. coli* [94], *Hbt. salinarum* [95]
CheB demethylates MCP	*B. subtilis* [123], *E. coli* [122], *Hbt. salinarum* [95]
CheC dephosphorylates CheY	*B. subtilis* [82]
CheD deamidates MCP	*B. subtilis* [93], *T. maritima* [85]
CheD demethylates MCP	*T. maritima* [85]
CheR methylates MCP	*B. subtilis* [124], *E. coli* [122], *Hbt. salinarum* [95]
CheX dephosphorylates CheY	*B. burgdorferi* [148]
CheZ dephosphorylates CheY	*E. coli* [149]
FliY dephosphorylated CheY	*B. subtilis* [82]
