# Supplementary material for: The protein interaction network of a taxis signal transduction system in a Halophilic Archaeon
Source: BMC Microbiol. 2012 Nov 21;12:272. doi: 10.1186/1471-2180-12-272 (PMC3579733; doi:10.1186/1471-2180-12-272)
Supplement: Additional file 8 — CheA peptides identified in bait fishing experiments with CheW1 and OE4643R give no indication for different CheA subspecies. The complete CheA protein sequence is shown. Peptides in italics were identified with OE4643R and peptides shown underlined with CheW1. [file 1471-2180-12-272-S8.pdf]

1 *MDDYLEAFVR* EGEEHVTSLN NALLELESDP GNEEAMDEIF RTAHTLKGNF

51 GAMGFEDASD LAHAVEDLLD EMR*QGNLEVT* *SDR*MDRIFEG IDGIEACLDE

101 IQATGDVDR*D* *VTGTIESVRA* *VLDEVDGDGG* *SGTTTSSGDA* *GSPAGDGDVD*

151 *ATR*VVDADTI DAAEDPVYHI HIDMGDSQMK GVDGMFVLEE ATEAFDLLGA

201 EPSPDAINDG EYGDGFELVV ATPSDEVSDT VAAFPKLSDA TVTAVGDDEH

251 APDADSGTEA DASADDDADD AGTTADSGSS SGGSSAIDNT DTEIQSVRVD

301 VDQLDELHGL VEQLVTTRIK LRRGMEESDR *EVLDELDELD* *KITSSLQDTV*

351 *MDMRLVPMKK* *IVGKFPR*LVR DLAR*EQDKDI* *DFVVEGDDVE* *LDR*TILTEIS

401 DPLMHLLR*NA* *VDHGIEKPAV* *R*EDNGK*DREG* *TITLSAER*DR DHVLIQVR*DD*

451 *GAGIDHDTMR* *EK*AIEKGVKT REEVQDMPDD DVEDLVFHPG FSTNDEVTDV

501 SGR*GVGMDVV* *R*DTVTRLDGS VSVDSTPGEG TTFTMTLPVT VAIVK*VLFVE*

551 *SGGEEYGIPI* *KTVDEISR*MK SVKSVDGEEV ITYDETVYPL VR*LGDALNVP*

601 *DETRNGDGML* *VR*IRDSER*QV* *AVHCDDVR*GQ EEVVVKPFEG ILSGIPGLSG

651 AAVLGEGDVV TILDVATL
