# Supplementary material for: The protein interaction network of a taxis signal transduction system in a Halophilic Archaeon
Source: BMC Microbiol. 2012 Nov 21;12:272. doi: 10.1186/1471-2180-12-272 (PMC3579733; doi:10.1186/1471-2180-12-272)
Supplement: Additional file 9 — Observations characterizing protein complexes of the core signaling proteins. Preys identified with relatively high sequence coverage but a SILAC ratio close to one in one-step bait fishing and identified as interactors in two-step bait fishing (Additional file 4) were assumed to exchange. For the underlying data see Additional file 3 and Additional file 4. [file 1471-2180-12-272-S9.pdf]

Observations characterizing protein complexes of the core signaling proteins.

	Observation
1	one-step bait fishing with CheA: CheW1 exchanged
2	one-step bait fishing with CheA: PurNH static interactor
3	one-step bait fishing with CheA: Htrs static interactors
4	one-step bait fishing with CheA: OE4643R static interactor
5	two-step bait fishing with CheA: CheW1 interactor
6	two-step bait fishing with CheA: Htrs not interactors
7	two-step bait fishing with CheA: PurNH not interactor
8	two-step bait fishing with CheA: OE4643R not interactor
9	one-step bait fishing with CheW1: CheA exchanged
10	one-step bait fishing with CheW1: PurNH exchanged
11	one-step bait fishing with CheW1: Htrs static interactors
12	two-step bait fishing with CheW1: CheA interactor
13	two-step bait fishing with CheW1: PurNH interactor
14	two-step bait fishing with CheW1: Htrs interactors
15	one-step bait fishing with CheW2: Htrs exchanged
16	one-step bait fishing with CheW2: CheA not interactor
17	two-step bait fishing with CheW2: Htrs interactors
18	two-step bait fishing with CheW2: CheA not interactor
19	one-step bait fishing with OE4643R: CheA static interactor
20	one-step bait fishing with OE4643R: Htrs not interactors
21	one-step bait fishing with OE4643R: CheW1 not interactor
22	one-step bait fishing with OE4643R: PurNH not interactor
23	two-step bait fishing with OE4643R: CheA interactor
24	two-step bait fishing with OE4643R: Htrs not interactors
25	two-step bait fishing with OE4643R: CheW1 not interactor
26	two-step bait fishing with OE4643R: PurNH not interactor
27	one-step bait fishing with PurNH: CheA static interactor
28	one-step bait fishing with PurNH: Htrs static interactors
29	two-step bait fishing with PurNH: none of the here discussed proteins interactors

Preys identified with relatively high sequence coverage but a SILAC ratio close to one in one-step bait fishing and identified as interactors in two-step bait fishing were assumed to exchange.
