# Supplementary material for: The protein interaction network of a taxis signal transduction system in a Halophilic Archaeon
Source: BMC Microbiol. 2012 Nov 21;12:272. doi: 10.1186/1471-2180-12-272 (PMC3579733; doi:10.1186/1471-2180-12-272)
Supplement: Additional file 10 — Primers used in this study. [file 1471-2180-12-272-S10.pdf]

Primers used in this study

Primer	Sequence
OE1428F_fo	CACCGTGCTGAGTTCCAGCAGCGACGC
OE1428F_re	CGCGCGAGTGAACGCGTCGAGTC
OE1620R_fo	CACCATGTCCACCATCGCTGGTCTGG
OE1620R_re	GTCGTGGCGGAACGCCCGGTGG
OE2374R_fo	CACCATGTCCGATGACGAGACGGAC
OE2374R_re	TACGAGGGCGTCGGGTTCGAC
OE2401F_fo	CACCGTGCCATCGCTGTACGGGCTGG
OE2401F_re	CGTTTTTCCGCCCAGCTTCGAGATC
OE2402F_fo	CACCATGAGCGAGTCAGAGTACAAGATAG
OE2402F_re	TTCCTCGTTGATCGCTTCGCTGGC
OE2404R_fo	CACCATGTCGGAATCAGCAATCGCAGAC
OE2404R_re	GAGGTTGACGTCCTCGATGTGTTC
OE2406R_fo	CACCTTGACTGACTTCCAAACCCTG
OE2406R_re	CGTGTCAGCGACCCGACTGTAG
OE2408R_fo	CACCGTGACGATCCGCGTTGGCGTG
OE2408R_re	AATTACGTGCACCTCGGCATC
OE2410R_fo	CACCATGCGTGTCGATCTCGACGC
OE2410R_re	CGACTCCTCACTCGCGTCGG
OE2414R_fo	CACCATGAGCACAATGATCGACATTC
OE2414R_re	GATACTGTTGATCATCGAGAC
OE2415R_fo	CACCATGGACGACTACCTCGAAGCGTTCG
OE2415R_re	CAGCGTAGCCACGTCCAGGATGGTC
OE2416R_fo	CACCATGACAGAGGCACTGGTGGTC
OE2416R_re	CGTCGTCCTCCGTATCGAATC
OE2417R_fo	CACCATGGCGAAGCAGGTCTTACTGGTC
OE2417R_re	TGCGGTGAGCACGTCCGAAATAGCG
OE2419R_fo	CACCATGAACCTCGAAGACGCCGAC
OE2419R_re	GACATGATTGTCGATGGTCTC
OE3280R_fo	CACCATGAGTGCCACGATCGAGCTG
OE3280R_re	CGAGAGGTCGTTGAGCAGCGAC
OE4643R_fo	CACCATGAACGCCGACATCGACGCGGTG
OE4643R_re	CTCATCTCCCATCACCTCCCGGAT
M13F (-20)	GTAAAACGACGGCCAG
M13R (-26)	CAGGAAACAGCTATGAC
