# Supplementary material for: The protein interaction network of a taxis signal transduction system in a Halophilic Archaeon
Source: BMC Microbiol. 2012 Nov 21;12:272. doi: 10.1186/1471-2180-12-272 (PMC3579733; doi:10.1186/1471-2180-12-272)
Supplement: Additional file 11 — Proteins considered to be contaminants. [file 1471-2180-12-272-S11.pdf]

Proteins considered to be contaminants.

Protein	Reason
OE1275F	Highly promiscuous, involved in protein degradation (probably due to misfolded bait)
OE1736R	Promiscuous, involved in protein folding (probably due to misfolded bait)
OE1737R	Binds to CBD
OE2201F	Binds to cellulose column
OE2205F	Binds to cellulose column
OE2206F	Binds to cellulose column
OE2296F	Highly promiscuous, involved in protein degradation (probably due to misfolded bait)
OE2998R	Binds to CBD
OE3642F	Binds to CBD
OE3925R	Promiscuous, involved in protein folding (probably due to misfolded bait)
OE4122R	Promiscuous, involved in protein folding (probably due to misfolded bait)
OE4674F	Binds to CBD
